# The challenging diagnosis of food protein-induced enterocolitis syndrome: A case report series

**DOI:** 10.3389/fped.2022.913278

**Published:** 2022-09-20

**Authors:** Caiyan Zhao, Ling Chen, Jinzhi Gao

**Affiliations:** Department of Pediatrics, Tongji Hospital, Tongji Medical College, Huazhong University of Science and Technology, Wuhan, China

**Keywords:** food protein-induced enterocolitis syndrome (FPIES), cow’s milk protein allergy (CMPA), neonate, IgE, atypical

## Abstract

Food protein-induced enterocolitis syndrome (FPIES) is a type of non-immunoglobulin E (IgE)-mediated food allergy. However, in addition to vomiting and diarrhea, IgE-mediated skin or respiratory symptoms may be comorbidities in some patients with FPIES. We described four unusual cases of neonates with FPIES, whose clinical presentations were variable and misleading. All patients experienced vomiting, diarrhea or other gastrointestinal symptoms, and three of them developed IgE-mediated food allergy. Case 1 was admitted to the hospital with convulsions and then developed severe sepsis and necrotizing enterocolitis (NEC)-like appearance. Case 2 was wrongly diagnosed with Stevens–Johnson syndrome due to a severe extravasation rash of the skin and mucous membranes and a systemic inflammatory response. There was unexplained cholestasis in case 3, which might be attributed to food allergy. Asymptomatic elevation of C-reactive protein was the only hint at early-stage FPIES in case 4. Moreover, there were increased serum food-specific IgG values in three of the above cases. After eliminating the offending food, all of the above clinical manifestations rapidly improved in the four cases; thus, we believe that the most correct diagnosis in the described four cases was FPIES. This case report series should further draw clinicians’ attention to FPIES with variable and atypical symptoms. The usefulness of IgG levels in identifying the presence of FPIES is uncertain.

## Introduction

Cow’s milk protein allergy (CMPA), which is the most common food allergy within the first year of life, can be broadly categorized into either IgE-mediated or non-IgE-mediated processes. Signs and symptoms of IgE-mediated CMPA reactions often involve urticaria and angioedema, respiratory tract and gastrointestinal symptoms, or even anaphylaxis, which have rapid onset, typically beginning within hours after ingestion. Unlike IgE-mediated CMPA, non-IgE-mediated processes exhibit isolated acute or chronic gastrointestinal symptoms, such as vomiting, diarrhea and bloody stools. Among no-IgE-mediated CMPA-associated gastrointestinal disorders, food protein-induced enterocolitis syndrome (FPIES) has the lowest incidence rate but represents the most severe condition (1). Acute FPIES is commonly characterized by repetitive emesis, beginning approximately 1–4 h after food ingestion, lethargy, pallor, diarrhea, dehydration, metabolic acidosis, hypotension and shock, among other symptoms (2). Chronic FPIES presents with intermittent emesis, watery or bloody stools, and failure to thrive after regular or repeated ingestion of the triggering food (3). Due to the absence of specific confirmatory tests, FPIES is often misdiagnosed or undiagnosed. Here, we described four unusual cases of neonates with FPIES presenting with peculiar clinical features, such as convulsions, a rash misdiagnosed as Stevens-Johnson syndrome, cholestasis and asymptomatic C-reactive protein (CRP) elevation. Given that all four patients experienced gastrointestinal symptoms and failure to thrive, and that the symptoms were relieved after allergen avoidance, we believe that FPIES was the most correct diagnosis in these cases. Subsequently, the mothers avoided possible allergens, including milk, eggs, soy, beef and mutton, and the infants who were fed with breast milk did not experience a new symptom onset.

International guidelines include as acute FPIES diagnostic criteria the absence of IgE-mediated symptoms and undetectable serum food-specific or total IgE antibodies and skin prick test (4). However, patients with atypical FPIES develop positive skin test and/or food-specific IgE (5), and previous studies showed that 2–30% of patients with PFIES have evidence of IgE positivity (1, 6, 7). Further, patients with atypical FPIES have a predisposition for IgE-mediated allergic skin or respiratory symptoms, including atopic dermatitis and asthma (8). Our case series also highlighted that the diagnosis of FPIES cannot be simply ruled out based on IgE-mediated allergic skin or respiratory symptoms. Although three of our cases showed high levels of IgG, which gave us clues to the diagnosis of PFIES, the usefulness of IgG levels in identifying the presence of FPIES is still uncertain.

## Case presentations

### Case 1

**FIGURE 1:**
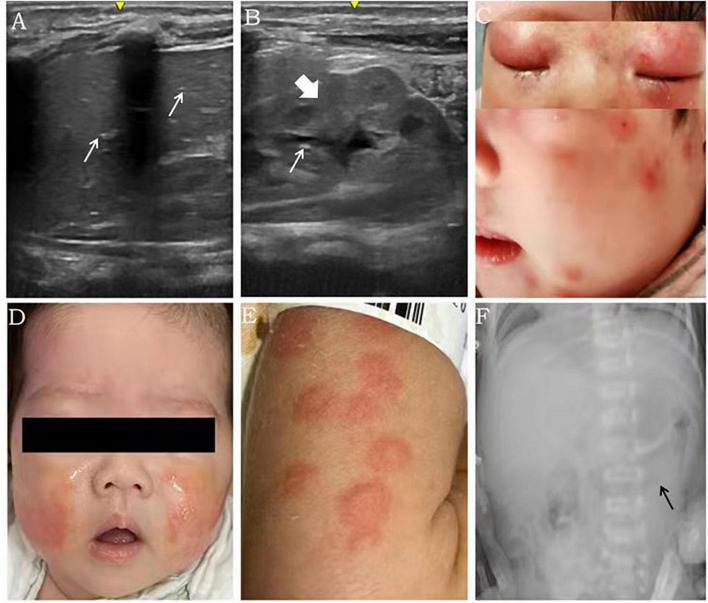
Imaging findings and rash features in patients with FPIES. Scattered echoes of gas in the anterior right lobe [**(A)** white arrow], diffuse bowel wall thickening (with a thickness of 3.3 mm) [**(B)**, coarse arrow] and scattered echoes of gas in parts of the intestinal wall [**(B)**, white arrow] were detected using ultrasound in case 1. Numerous red exudative papules fused and distributed all over the body, including the face, were observed in case 2 **(C)**. Both the upper and lower eyelids were red and swollen with little ulceration **(C)**. The rash in case 2 improved gradually after avoiding milk ingestion **(D)**. Urticaria was distributed all over the body in case 3 **(E)**. Distended loops of intestine were seen in X-ray in case 4 [**(F)**, black arrow)].

A boy was born to a 28-year-old mother at 38 weeks gestation by vaginal delivery with a birth weight of 3,750 g. The membranes (amniotic sac) ruptured 24 h before the delivery. The newborn was fed with both breast milk and infant formula. Diarrhea developed on the 7th day of life, and the newborn was transferred to a neonatal intensive care unit (NICU) at the age of 17 days because of intermittent convulsions for 7 days. The serum free calcium concentration was 0.664 mmol/L (2.25–2.75 mmol/L), other serum electrolyte concentrations, renal function, and parathyroid function and the findings from the patient’s brain MRI, cerebrospinal fluid analysis and electroencephalogram were within normal ranges. The convulsions disappeared after calcium supplementation. On the second day in the NICU, the infant developed fever, abdominal distension, diarrhea and bloody stools. The laboratory results showed normal total white cell count, eosinophil cell count and platelet count, but high levels of CRP (178.9 mg/L, normal: <0.5 mg/L), tumor necrosis factor-α (TNF-α) (73.5 pg/mL), interleukin-6 (1,399 pg/mL) and interleukin-8 (1,824 pg/mL). Sepsis was suspected, and intravenous antibiotics (including ceftriaxone, teicoplanin, and meropenem) were prescribed, although the blood and stool cultures were negative. Since abdominal ultrasound revealed weakened intestinal motility, bowel wall thickening, pneumatosis intestinalis and intrahepatic pneumatosis (Figures 1A,B), necrotizing enterocolitis (NEC) was suspected, and the feedings were discontinued. Then, the fever and gastrointestinal symptoms disappeared, but the blood tests showed methemoglobinemia and elevated white blood cell count. What’s more, though food-specific antibody determination showed that the total IgE and cow’s milk-specific IgE were negative, the cow’s milk-specific IgG was higher than 400 U/mL (negative: <50 IU/mL). Thereafter, FPIES was considered, and feedings with amino acid-based formula (AAF) and breast milk were started. Three days later, all symptoms disappeared and all laboratory tests returned normal. Regular formula was introduced once when the baby was 2-month-old, and fever, diarrhea and emesis quickly reappeared within few hours. When the patient was almost 7-month-old, a skin prick test showed eggs (2+) and milk (-), although the baby had persistently been fed with AAF and breast milk.

### Case 2

A girl was born to a 38-year-old mother at 39 weeks gestation by Cesarean section with a birth weight of 3,000 g. The membranes ruptured 4 h before the delivery. The infant was fed with both breast milk and infant formula 1 h after the birth. A perianal rash appeared on the 8th day of life, intermittent vomiting and diarrhea developed on the 28th day of life. At the age of 1 month, the infant was admitted into a hospital for fever and severe rash, which manifested as a remarkable perianal rash with herpes and numerous red papules fused all over the body, including the face (Figure 1C). Both the upper and lower eyelids were red and swollen with little ulceration (Figure 1C). The laboratory results showed a total white cell count of 31.65 × 10^9^/L, platelet count of 656 × 10^9^/L and neutrophil ratio of 51.2%. The CRP level, erythrocyte sedimentation rate and TNF-α level were increased to 73.72 mg/L, 120 mm/h and 18.9 pg/mL, respectively. Moreover, the fecal occult blood test was positive. Although there were no abnormal findings in the patient’s cerebrospinal fluid and blood culture, cefoperazone was started due to suspicion of sepsis. A few days later, the total white cell count and CRP level still continued to increase, and cefoperazone was replaced by vancomycin, meropenem, ganciclovir and fluconazole. The whole body skin rash intensified, and Stevens-Johnson syndrome diagnosis was suspected. The infant was admitted for intravenous methylprednisolone treatment after EB virus, rubella, cytomegalovirus, toxoplasma and herpesvirus were ruled out. Subsequently, the fever disappeared, and the diarrhea and rash improved, but the total white cell count, platelet count and CRP level were still abnormal, which prompted the patient’s transfer to our care center for further workup on the 49th day of life. The brother of the infant had atopic cough. The determination of specific antibodies revealed negative total IgE and food-specific IgE, while the cow’s milk-specific IgG was elevated above 400 U/mL. FPIES was considered, and feeding of AAF and breast milk was implemented. The total white cell count and CRP level normalized, vomiting and diarrhea improved, and the rash subsided 5 days later (Figure 1D).

### Case 3

A preterm baby was born at 33 weeks gestation by Cesarean section with a birth weight of 1,450 g to a 31-year-old mother with severe preeclampsia and gestational diabetes. The delivery course was unremarkable. Preterm formula (3 ml every 3 h) was initiated after the birth. Remarkable abdominal distension presented within 5 days of life, and frequent emesis occurred after 3 days of life; subsequently, the feedings were discontinued. The diagnoses feeding intolerance and early-onset FPIES were considered. Trophic feeding was restarted with extensively hydrolyzed formula (eHF) from 9 days of life and advanced at a speed of 20 ml/kg/day. The laboratory results showed normal total white cell count, platelet count and CRP levels. The parenteral nutrition was discontinued on the 18th day of life, and preterm formula was introduced again instead of eHF. Seventeen days later, abdominal distension, emesis, diarrhea, fever and a rash (Figure 1E) presented in succession. The CRP level increased to 12 mg/L. The total IgE was positive (1,583.5 IU/mL), whereas the IgEs specific for cow’s milk, beef and mutton were strongly positive. Based on the results, the preterm formula was replaced by eHF. Then, the CRP level normalized, and the rash and gastrointestinal symptoms improved. Notably, the direct bilirubin level increased from 22.3 μmol/L (total bilirubin 138.2 μmol/L) on the 7th day of life to 41.7 μmol/L (total bilirubin 88.1 μmol/L) on the 38th days of life. The liver function was normal throughout the hospital stay, and a cytomegalovirus infection was excluded. The direct bilirubin level reduced to 25.5 μmol/L on the 52nd day of life during the follow up, and the infant was fed with eHF.

### Case 4

**FIGURE 2:**
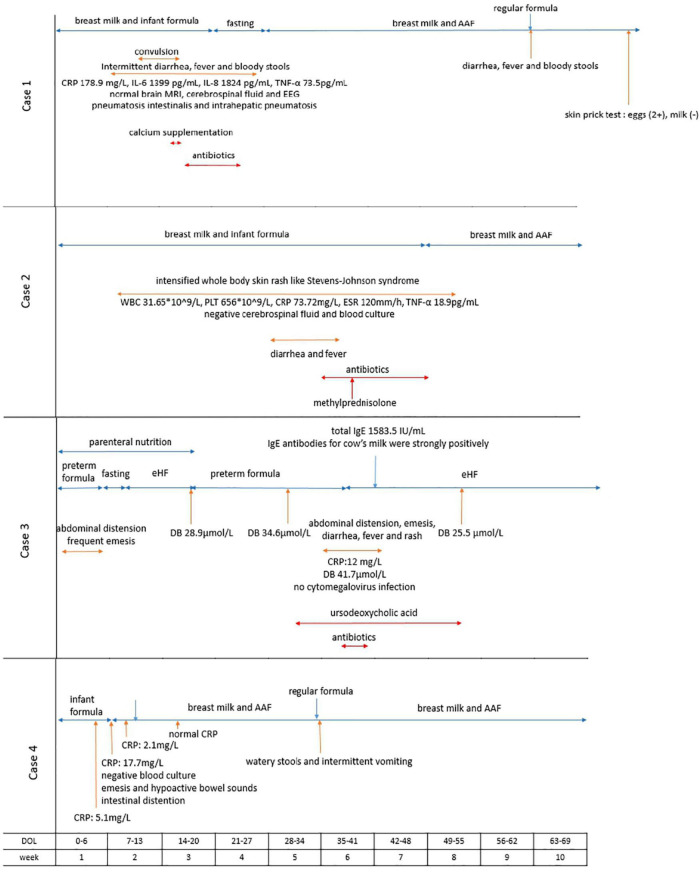
Timeline of symptom development, serum-specific antibodies tests, dietary changes and other laboratory findings in the cases. AAF, amino acid-based formula; CRP, C-reactive protein; IL-6, interleukin-6; IL-8, interleukin-8; TNF-α, tumor necrosis factor-α; EEG, electroencephalogram; ESR, erythrocyte sedimentation rate; eHF, extensively hydrolyzed formula.

A boy was born to a 30-year-old mother at 40 weeks gestation by a vaginal delivery with a birth weight of 2,860 g. He was admitted into a hospital as a high-risk infant, because the group B *Streptococcus agalactiae* (GBS) screening of his mother was positive. Regular formula was initiated soon after the birth. The CRP level increased to 5.1 mg/L on the 4th day of life, but the boy was asymptomatic. Meanwhile, blood culture and next-generation sequencing results indicated no GBS infection in the boy. The CRP level gradually increased to 17.7 mg/L on the 7th day of life, while the procalcitonin level was normal. In addition, emesis and hypoactive bowel sounds appeared on the same day. An X-ray suggested intestinal distention (Figure 1F). Although an allergy antibody test indicated that the IgE levels were negative, the cow’s milk-specific IgG was 118 U/mL, and FPIES was considered based on the clinical feature. Then, the regular formula was replaced by AAF. On the next day, the patient was discharged from the hospital, after the CRP level decreased to 2.1 mg/L. The boy was fed with AAF and breast milk after the discharge. One week later, the CRP level normalized. In the outpatient follow-up, the regular formula was introduced again when the baby was 1 month old, and watery stools and intermittent vomiting appeared within 24 h (Timeline and summary of case series are shown in Figure 2 and Table 1).

**TABLE 1:** Summary of case series.

Case	Case 1	Case 2	Case 3	Case 4
Age at onset	7 days	8 days	35 days	7 days
Diet before diagnosis	Breast milk and infant formula	Breast milk and infant formula	Preterm formula	Regular formula
Latency between old diet and symptom onset	7 days	8 days	17 days	7 days
Symptoms of the digestive tract	Abdominal distension, diarrhea and bloody stools	Intermittent vomiting and diarrhea	Abdominal distension, emesis and diarrhea	Emesis and hypoactive bowel sounds
Other symptoms	Convulsions and fever	Severe rash like Stevens-Johnson syndrome and fever	Rash, fever and cholestasis	No
White blood cell count (×10^9^/L)	20.27	31.52	13.86	17.52
Eosinophil count (%)	5.1	3.0	18.2	4.0
Platelet count (×10^9^/L)	392	656	336	334
CRP (mg/L)	178.9	73.72	12	17.7
Evidence of IgE positive				
Elevated sIgE	No	No	Yes	No
SPT	Positive to egg	Not performed	Not performed	Not performed
Elevated cow’s milk-specific IgG	Yes	Yes	No	Yes
Abdominal ultrasound or X-ray	Weakened intestinal motility, bowel wall thickening, pneumatosis intestinalis and intrahepatic pneumatosis	Normal	Normal	Intestinal distention
Antibiotics	Yes, ceftriaxone, teicoplanin and meropenem	Yes, cefoperazone, vancomycin, meropenem, ganciclovir and fluconazole.	No	No
Family history	No	Yes	No	No

CRP, C-reactive protein; SPT, skin prick test.

## Discussion

Most patients with CMPA develop symptoms between 2 and 6 weeks of age. The median age of onset is 7 days in neonate cases (9), and even a case with a fetal onset has been reported (10). However, variable and atypical clinical manifestations and the lack of confirmatory tests delay the diagnosis of CMPA, which is accompanied by extensive investigations, unnecessary treatments and prolonged hospitalizations. Healthcare providers should raise the awareness of CMPA. According to its clinical manifestations, FPIES is a serious CMPA subtype, and an early diagnosis can improve the survival rate of affected newborns.

### The spectrum of clinical presentations of FPIES is broad, and IgE-mediated skin or respiratory symptoms may be comorbidities

International guidelines have proposed accurate diagnostic criteria for acute FPIES, typical clinical symptoms are repetitive vomiting, diarrhea and possible dehydration or lethargy within few hours following the ingestion of the suspected food (4). The chronic FPIES is characterized by frequent watery diarrhea, intermittent vomiting, weight loss, and failure to thrive. Although FPIES has been considered to be triggered by non-IgE-mediated food hypersensitivity (11, 12), the absence of IgE-mediated skin or respiratory symptoms and/or positive IgE results is not necessary to diagnose FPIES (13–15). The most important diagnostic criteria for FPIES are allergic symptoms that improve within few days after the elimination of the offending food and recrudesce after its reintroduction. Actually, approximately 2–12%, 2–30%, and 20–40% of the patients with FPIES have IgE-mediated symptoms, positive levels of IgEs specific to the FPIES-inducing food and sensitization to other foods, respectively (1, 6, 7). Atopic comorbidity was higher in patients with FPIES compared with healthy children, including atopic dermatitis, allergic rhinitis and IgE-mediated food allergy (16). Furthermore, one investigation suggested that 35% of patients with FPIES having positive IgE results can convert to IgE-mediated CMPA (16). In our case series, case 1 was diagnosed as FPIES using an oral food challenge (OFC). The IgE level was negative during the early stage; non-IgE mediated symptoms, such as fever, diarrhea and emesis, developed after the reintroduction of the regular formula and were not accompanied by IgE-mediated symptoms; however, the skin prick test was positive to egg. In case 2, FPIES was suspected based on the clinical manifestations, with extremely severe skin masses and blisters, which might be IgE-mediated, although the IgE level was negative. Case 3 presented with a remarkable rash accompanied by extremely increased IgE levels. There is no specific test for FPIES, but the skin prick test and/or serum food-specific antibody test may be performed as a part of the evaluation.

### The clinical presentations of chronic FPIES are variable, atypical and misleading

The spectrum of clinical presentations of FPIES is broad. In addition to decreased appetite, intermittent emesis, diarrhea, abdominal distension, dehydration and failure to thrive, it may also include metabolic acidosis, fever and lethargy. In addition, the main symptoms of FPIES are age-related. Patients who become symptomatic within 2 months after birth are more likely to have diarrhea and bloody stools, while others are more likely to develop vomiting (17, 18). Therefore, FPIES may be easily misdiagnosed as other diseases, such as gastroenteritis, sepsis, intussusception, NEC, surgical abdomen or inborn errors of metabolism. Especially during the neonatal period, NEC may be considered when a newborn has bloody stools (19, 20). Leukocytosis and the intestinal motility outside the lesion area may be the differentiations of FPIES and NEC (21). In case 1, pneumatosis intestinalis and intrahepatic pneumatosis were detected using ultrasound, which have always been regarded as signs of NEC. The patient even developed abdominal distention and abdominal rumbling sound weakening and was misdiagosed as NEC. However, considering that the patient was a full-term newborn with no risk factors for developing NEC and had leukocytosis and high CRP levels but normal PCT levels, FPIES was suspected. After the elimination of allergens, the symptoms disappeared and the diagnosis of FPIES was confirmed. Furthermore, many other misleading symptoms were also observed in our case series. Case 1 was admitted to the NICU because of convulsions due to hypocalcemia induced by prolonged diarrhea. Thus, for the patient admitted to a hospital with convulsions, medical record and physical examination are extremely important. Case 2 was misdiagnosed as Stevens-Johnson syndrome because of the intensified whole body skin rash and systemic inflammatory response syndrome. Furthermore, since case 1 and case 2 developed fever, leukocytosis, and elevated CRP levels, sepsis was suspected and unnecessarily prolonged antibiotics were used, although the patient’s blood cultures were negative. The differentiations of FPIES and sepsis may include elevated platelet counts (22), a rapid recovery from shock through rehydration, and family history of allergies. Both case 1 and case 2 had high platelet levels. Case 1 quickly recovered from lethargy and shock after rehydration. Case 2 had family history of allergies. The unexplained cholestasis in case 3 could be attributed to FPIES. Inflammation caused by FPIES may lead to bile duct edema and subsequent blockage of direct bilirubin discharge. After the preterm formula was replaced by eHF, the direct bilirubin level gradually declined. Additionally, case 4 was also diagnosed with FPIES using OFC. The patient was asymptomatic with only elevated CRP levels early in the course. With the progress of the disease, emesis, hypoactive bowel sounds and intestinal distention developed. An elevated CRP value may be the only hint during the early stage of FPIES, whereas gastrointestinal symptoms occur gradually with increasing CRP levels. An OFC is recommended to make an accurate diagnosis of FPIES. Actually, the diagnosis can be made when various symptoms disappear after the elimination of the offending food, and other etiologies are excluded (23).

### The usefulness of IgG levels in identifying the presence of FPIES is uncertain

IgE-mediated CMPA is mediated by the type I hypersensitivity immune response, while the mechanisms of non-IgE-mediated CMPA are unclear. Cellular and humoral immunity may both be implicated. The intestinal epithelial cells take certain types of food particles up, and the immune system sequentially recognizes them as harmful substances and produces an excessive protective immune response and specific IgGs against the implicated food. The immune complexes comprising specific IgGs and the implicated food particles could cause a type III allergic reaction (24). After the elimination of the suspected allergens from the diet, based on the results for the specific IgGs for the implicated foods, the clinical symptoms improve significantly with the decrease of IgG levels and IgG4 subtype titers (25). It was suggested that elevated serum food-specific IgG levels may indicate FPIES induced by a specific allergen. We detected high cow’s milk-specific IgG levels in cases 1, 2, and 4, while the total IgE and specific IgE levels were undetectable. However, many scholars believe that elevated IgG levels only indicate that the human body is more exposed to a certain food, not that it is allergic to a certain food. Most of studies about the relationship between FPIES and quantification of IgG find low levels of cow’s milk (CM) specific IgG and IgG4 in patients with CM-FPIES versus those tolerating CM (26–29). So most national and international allergy societies do not recommend elevated serum food-specific IgG levels as an indicator for diagnosis of FPIES (4, 30, 31).

Recently, the incidence rate of FPIES has been increasing, but an accurate diagnosis is still challenging. A misdiagnosis would lead to immune dysfunction and growth retardation and aggrandize unnecessary treatment inspection costs. The clinical presentation of FPIES is variable, atypical and misleading. No matter whether there are IgE-mediated skin or respiratory symptoms, once intermittent but progressive vomiting and/or diarrhea occurs in an infant, particularly in an infant who has been fed with regular cow’s formula, FPIES should be considered. Most of all, clinicians should always pay attention to allergies.

## Data availability statement

The original contributions presented in this study are included in the article/supplementary material, further inquiries can be directed to the corresponding author.

## Ethics statement

The studies involving human participants were reviewed and approved by Medical Ethics Committee, Tongji Hospital, Tongji Medical College, Huazhong University of Science and Technology.

## Author contributions

CZ, LC, and JG contributed to conception and design of the study, wrote sections of the manuscript. CZ wrote the first draft of the manuscript. All authors contributed to manuscript revision, read, and approved the submitted version.
